# Efficacy and safety of high-dose chemotherapy as the first or subsequent salvage treatment line in patients with relapsed or refractory germ cell cancer: an international multicentric analysis

**DOI:** 10.1016/j.esmoop.2024.103449

**Published:** 2024-05-13

**Authors:** C. Seidel, C. Schaefers, E.A. Connolly, A. Weickhardt, P. Grimison, V. Wong, U. De Giorgi, M. Hentrich, S. Zschäbitz, S. Ochsenreither, B. Vincenzi, C. Oing, C. Bokemeyer, N. Engel, W. Alsdorf, B. Tran

**Affiliations:** 1 Department of Oncology, Hematology and Stem Cell Transplantation with Division of Pneumology, University Medical Center Hamburg-Eppendorf, Hamburg, Germany; 2 Department of Medical Oncology, Chris O’Brien Lifehouse, Sydney; 3 Olivia Newton-John Cancer and Wellness Centre, Austin Health, Heidelberg; 4 Walter and Eliza Hall Institute of Medical Research, Melbourne, Australia; 5 Department of Medical Oncology, IRCCS Istituto Romagnolo per lo Studio dei Tumori (IRST) “Dino Amadori”, Meldola, Italy; 6 Department of Hematology and Oncology, Red Cross Hospital Munich, Ludwig Maximilian University of Munich, Munich; 7 Department of Medical Oncology, National Centre for Tumor Diseases (NCT), University Hospital Heidelberg, Heidelberg; 8 Charité Comprehensive Cancer Center, Charité - Universitätsmedizin Berlin, Berlin, Germany; 9 Department of Medical Oncology, Campus Bio Medico University of Rome, Rome, Italy; 10 Translational and Clinical Research Institute, Centre for Cancer, Newcastle University, Newcastle upon Tyne, UK; 11 Mildred Scheel Cancer Career Centre HaTriCs4, University Cancer Center Hamburg, University Medical Center Hamburg-Eppendorf, Hamburg, Germany; 12 Center for Cellular Immunotherapies, Department of Pathology and Laboratory Medicine, University of Pennsylvania Perelman School of Medicine, Philadelphia, USA; 13 Department of Medical Oncology, Peter MacCallum Cancer Centre, Melbourne, Australia

**Keywords:** relapse, refractory germ cell tumor, salvage chemotherapy, conventional-dose salvage regimens, high-dose chemotherapy

## Abstract

**Background:**

In relapsed or refractory (RR) metastatic germ cell cancer (GCC), high-dose (HD) chemotherapy (CTX) plus autologous stem cell transplantation is considered the standard of care. Limited data exist regarding the efficacy of HD-CTX following conventionally dosed salvage regimens (CDRs). This analysis explores and contrasts the efficacy of HD-CTX as the first or subsequent salvage regimen.

**Patients and methods:**

Data were retrospectively collected to explore the efficacy of HD-CTX administered as the first (group A) or subsequent salvage CTX (group B) after a CDR. The primary endpoint was OS from the time of HD-CTX. Associations of survival, overall response rate (ORR), and toxicity with clinical characteristics were explored using stratified Kaplan–Meier and Cox regression models.

**Results:**

Overall, 283 patients with GCC were included from 11 international centers, with 159 patients (56%) in group A and 124 patients (44%) in group B. The first salvage treatment was administered between 1998 and 2022, with a median follow-up of 27.0 [standard deviation (SD) 46.2] months for group A and 17.0 (SD 48.5) months for group B. The median OS from HD-CTX treatment initiation was not reached in group A, compared with 25 months in group B (*P* = 0.00027), associated with 2- and 5-year OS rates of 74% and 63% (group A) versus 53% and 37% (group B), respectively. When administered as the first salvage treatment, HD-CTX was associated with a higher ORR (79% versus 60%; *P* = 0.013) and lower nonhematologic grade ≥3 toxicity rate (78% versus 97%; *P* < 0.001). Concerning risk factor analysis for the total cohort, the International Prognostic Factors Study Group score was the only independent predictor of OS in multivariable analysis (*P* = 0.006).

**Conclusions:**

When administered as the initial salvage treatment or after CDR, HD-CTX exhibits curative potential for patients with RR GCC. The efficacy and safety outcomes were more favorable when HD-CTX was conducted as the first salvage treatment line.

## Highlights

• HD-CTX demonstrates curative potential, whether administered as the first or subsequent salvage regimen.
• When used as the first salvage therapy, OS was superior with a 2-year OS rate of 74%.
• HD-CTX yielded a higher 79% ORR when administered as the initial salvage treatment.
• HD-CTX was linked to lower grade ≥3 toxicities as the initial salvage regimen.
• Whether administered as the first or subsequent salvage regimen, HD-CTX should always be considered.

## Introduction

Germ cell cancer (GCC) is the most common tumor type in young men to the age of 40, and its incidence has been increasing in recent years.^1^, ^2^, ^3^ Because of its excellent sensitivity to cisplatin-based chemotherapy (CTX) in combination with multimodal treatment approaches, cure rates of >90% can be achieved even with metastatic disease.^4^^,^^5^ However, ∼30% of patients experience relapse or progression despite previous platinum-based first-line treatment. In such cases, long-term remissions are still achievable by administering salvage CTX regimens, which may involve conventional-dose or high-dose (HD)-CTX followed by autologous stem cell transplantation (ASCT). Conventional-dose salvage regimens (CDRs) are often conducted with four cycles of paclitaxel, ifosfamide, and cisplatin (TIP), while HD-CTX frequently consists of two to three consecutive cycles of HD carboplatin and etoposide (CE) plus ASCT.^6^, ^7^, ^8^, ^9^ As there is limited and inconclusive evidence from prospective trials, observational multicenter data from the International Prognostic Factors Study Group (IPFSG) study cohort, comprising 1594 patients, suggest an ∼10% improvement in overall survival (OS) favoring HD-CTX over CDR.^10^ However, the role of different salvage treatment regimens remains undefined, and results of the prospective comparison of HD-CTX versus CDR as the first salvage therapy in the current TIGER trial (NCT02375204) are pending. Moreover, in some regions of the world, the use of HD-CTX could be restricted when significant costs meet resource limitations.

When patients progress or relapse after the first salvage treatment, two different scenarios may arise. Typically, patients who experience progression or recurrence after HD-CTX receive combination CTX regimens with palliative intent, such as gemcitabine in combination with oxaliplatin (GO) with or without paclitaxel [GO(P)].^11^, ^12^, ^13^ However, in the case of relapse or progression after CDR, the use of HD-CTX may still offer a potentially curative treatment option. This assumption is based on limited data regarding the effectiveness of HD-CTX after at least one prior line of CDR salvage treatment. A retrospective analysis by Einhorn et al.^8^ demonstrated an inferior efficacy of HD-CTX using HD-CE after prior CDR compared with HD-CE when applied as the first salvage treatment. However, HD-CE still showed remarkable activity with a long-term disease-free survival rate of 44.9% after at least one line of prior CDR salvage treatment. Similar results were confirmed by the Memorial Sloan Kettering Cancer Center (MSKCC) experience reported in Feldman et al.^9^

By contrast, Lorch et al.^14^ reported on a series of patients receiving HD-CTX after prior CDR salvage treatment achieving a 5-year OS rate of 17% only. However, for this purpose, data were collected from several different HD-CTX approaches, many of which cannot be now considered standard of care.

A multicenter, multinational observational cohort study was carried out for this analysis to assess the efficacy and safety of HD-CTX following progression or recurrence of at least one CDR, compared with its use as the initial salvage treatment regimen.

## Patients and methods

### Study population and inclusion criteria

Through international and multicentric collaborations, we established an observational cohort analysis within a comprehensive dataset of patients with relapsed or refractory (RR) GCC. Our real-world study aimed to evaluate the effectiveness and safety, considering OS, overall response rate (ORR), and toxicity of HD-CTX, when administered for progression or recurrence following at least one prior salvage treatment regimen in comparison to HD-CTX administered as the first salvage treatment. Data were collected using revised and harmonized case report forms, and statistical analysis was carried out to address the study objectives. Patients who received HD-CTX as the first salvage therapy were categorized as group A, whereas those who received HD-CTX in subsequent salvage treatment lines were categorized as group B. To be eligible for participation, patients with GCC had to relapse or progress after at least three to six cycles of platinum-based CTX as the initial treatment and received subsequent treatment with HD-CTX with ASCT. There were no limitations concerning cycles and types of HD-CTX regimens. The diagnosis of GCC, encompassing both seminoma and nonseminoma types, needed to be confirmed through histological examination at the local institution or based on conclusive clinical findings, such as the presence of testicular, retroperitoneal, or mediastinal masses; elevated serum tumor marker levels; and a typical pattern of metastases. Participation using anonymized clinical data was in line with the local ethical standards of each participating center.

### Outcome measurements and statistical analysis

The primary objective of this study was to compare the median OS, 2- and 5-year OS rates from the time of the first relapse, and the time from HD-CTX treatment initiation between group A and group B. The secondary objectives were examining differences in ORR, which includes both partial and complete remission, the incidence of nonhematological grade ≥3 toxicities [according to Common Terminology Criteria for Adverse Events (CTCAE) version 5.0],^15^ and treatment-related deaths. Moreover, the study evaluated the same primary objectives within subgroups stratified by potential prognostic variables, including the IPFSG risk category, the International Germ Cell Cancer Cooperative Group (IGCCCG) risk category,^16^ and age, from the time point of HD-CTX treatment initiation. Survival analysis was conducted using the Kaplan–Meier method, with survival estimates compared using the log-rank test and multivariable Cox regression analysis. The *t*-test was used to compare various patient characteristics with the levels of tumor biomarkers. A two-sided *P* value of <0.05 was considered statistically significant. All statistical analyses were carried out using R (version 4.3.2; R Foundation, Vienna, Austria), with the use of packages gtsummary (version 1.7.2), survival (version 3.5-7), and finalfit (version 1.0.7).

## Results

### Patient characteristics

This analysis included 283 eligible patients from 11 centers from 1996 to 2022 who received HD-CTX as either the first salvage treatment (second line) after relapse or progression following platinum-based first-line CTX (group A; *n* = 159 patients) or after at least one prior conventionally dosed salvage treatment CTX (group B; *n* = 124 patients). The first salvage treatment was administered between 1998 and 2022, with a median follow-up of 27.0 [standard deviation (SD) 46.2] months for group A and 17.0 (SD 48.5) months for group B. In terms of histology, 27 (17%) patients in group A and 19 (15%) patients in group B revealed pure seminoma, and 131 (83%) and 105 (85%) patients in groups A and B revealed nonseminomatous histology, respectively. At the last follow-up, 172 patients (61%) were considered alive and 111 dead (39%). The date of the first salvage treatment initiation was available in 262 patients (92%), while the date of salvage HD-CTX was available in 231 patients (82%). Concerning patient characteristics, the only significant difference between group A and group B was an older mean age in group A, with 31.9 years compared with 29 years in group B (*P* = 0.007). Additional baseline patient characteristics before the first salvage treatment are detailed in Table 1.

**Table 1:** Patient characteristics

Characteristic	Group A (*N* = 159)^a^	Group B (*N* = 124)^a^	*P*-value^b^
Age	31.9 (9.3); (25.9-38.3)	29.0 (9.6); (22.2-35.3)	0.007
Seminoma/nonseminoma			0.7
Seminoma	27 (17)	19 (15)	
Nonseminoma	131 (83)	105 (85)	
N/A	1	0	
IPFSG			0.3
Very low	4 (3.4)	9 (7.9)	
Low	17 (15)	22 (19)	
Intermediate	46 (39)	33 (29)	
High	29 (25)	30 (26)	
Very high	21 (18)	20 (18)	
Missing	42	10	
IGCCCG			0.7
Good	42 (27)	30 (31)	
Intermediate	33 (22)	23 (23)	
Poor	78 (51)	45 (46)	
Missing	6	26	
Extrapulmonary metastasis yes/no			0.6
No	78 (67)	72 (63)	
Yes	39 (33)	42 (37)	
N/A	42	10	
Follow-up time (months)	27.0 (46.2); (9.0-72.0)	17.0 (48.5); (9.0-40.3)	0.2
Missing	18	52	

IGCCCG, International Germ Cell Cancer Cooperative Group; IPFSG, International Prognostic Factors Study Group.

a Data are presented as median (standard deviation); (interquartile range) or *n* (%) or *n*.

b Wilcoxon rank-sum test; Pearson’s chi-square test; Fisher’s exact test.

### Treatment approaches

The median time from the first-line treatment to the initiation of the first salvage treatment was 7.51 months (mean 25.04 months; interquartile range 10.25 months). Among the patients in the total cohort, the first-line treatment consisted of bleomycin, etoposide, and cisplatin (BEP) in 240 (85%) patients; etoposide, ifosfamide, and cisplatin (VIP) in 23 (4%) patients; etoposide and cisplatin (EP) in six (2%) patients; and other regimens in 14 (5%) patients. Concerning HD-CTX therapies, treatment was administered with two to three consecutive cycles of HD-CE in 256 patients (90%). Five patients received only one cycle of HD-CE (2%). Further regimens were HD-VIP; HD-CE with cyclophosphamide (CEC); HD-CE with thiotepa (CET); and high-dose ifosfamide, etoposide, and carboplatin (HD-ICE; 8%). Patients of group A and group B received a median of 2 (range 2-7) and 3 (range 2-7) different treatment lines in total, respectively. In group B, HD-CTX treatment was administered as subsequent salvage therapy in the third line in 99 patients (80%), in the fourth line in 21 patients (17%), and in the fifth line in four patients (3%). In group A, further treatment in cases of recurrence or progression after HD-CTX as the first salvage treatment is documented in 31 cases (19%), using GO and GO(P) and TIP (see Supplementary Appendix, available at https://doi.org/10.1016/j.esmoop.2024.103449).

### Survival and response analysis

**Figure 1:**
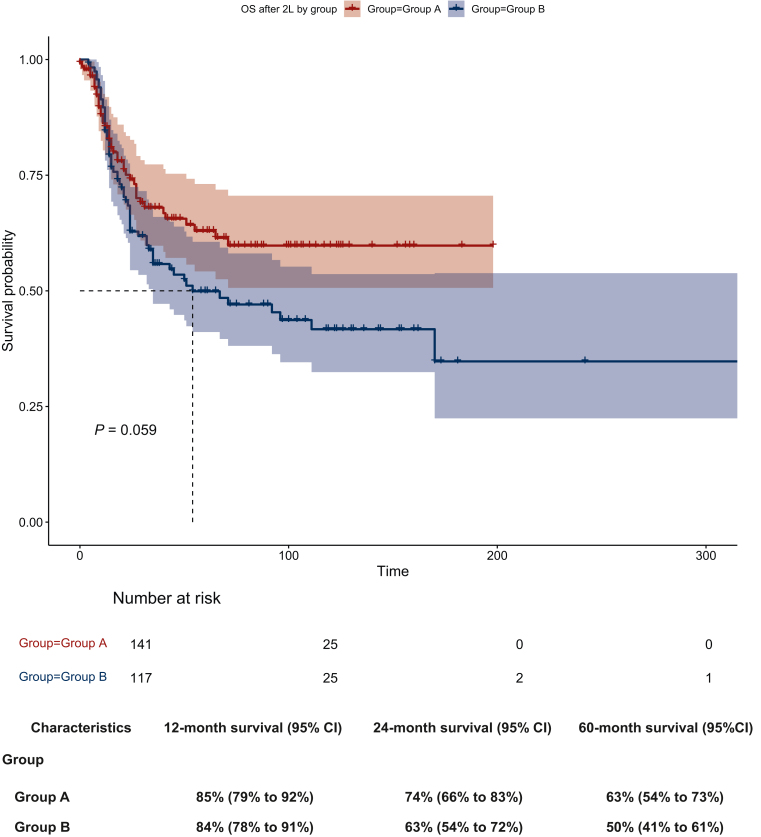
**Overall survival from the first salvage treatment initiation by group A versus B (*x*-axis time in days) with corresponding survival rates.** OS, overall survival.

**Figure 2:**
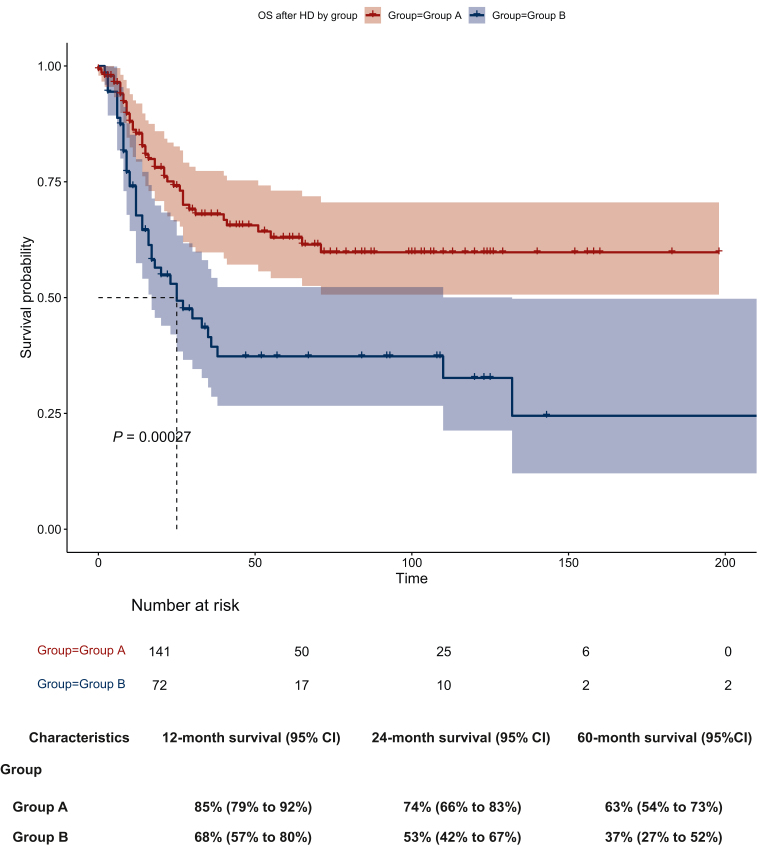
**Overall survival from high-dose (HD) chemotherapy treatment initiation by group (*x*-axis time in days) with corresponding survival rates.** OS, overall survival.

The median OS from the time of the first salvage treatment initiation was not reached in group A compared with 54 months in group B, without reaching significance (*P* = 0.059; Figure 1). The 2- and 5-year OS rates from the initiation of the first salvage treatment were 74% (group A) versus 63% (group B) and 63% (group A) versus 50% (group B), respectively (Figure 1). The median OS from HD-CTX treatment initiation was not reached in group A, compared with 25 months in group B (*P* = 0.00027). The 2- and 5-year OS rates from the initiation of HD-CTX were 74% (group A) versus 53% (group B) and 63% (group A) versus 37% (group B), respectively (Figure 2). The ORR following HD-CTX was 79% in group A and 60% in group B, showing a statistically significant difference (*P* = 0.013). In groups A and B, recurrent disease or progression after HD-CTX was associated with a 2-year OS rate of 18% in group A and 23% in group B, respectively.

### Risk factor analysis

The prognostic impact of the IPFSG and IGCCCG scores and age were examined for the time point of HD-CTX treatment for the entire cohort. Concerning OS, both IGCCCG score [good versus poor; *P* = 0.006; hazard ratio (HR) 2.34] and IPFSG risk groups (very low versus very high; *P* = 0.003; HR 21.04) were significant prognostic factors in the univariate analysis. In the multivariate analysis, the IPFSG score was an independent predictor of OS (*P* = 0.006; HR 22.20; Table 2). There were no statistical differences in OS for age. The results of the univariate analysis are described in Table 2.

**Table 2:** Results of univariate and multivariate analyses of overall survival from the start of high-dose chemotherapy for the total cohort

Dependent	All	HR (univariate)	HR (multivariate)
Age			
Mean (SD)	31.7 (9.5)	0.98 (0.96-1.01); *P* = 0.165	1.00 (0.97-1.03); *P* = 0.867
IPFSG			
Very low	13 (5.6)	—	—
Low	39 (16.9)	3.80 (0.49-29.74); *P* = 0.203	3.20 (0.39-26.17); *P* = 0.278
Intermediate	79 (34.2)	4.78 (0.64-35.77); *P* = 0.128	5.02 (0.60-42.06); *P* = 0.137
High	59 (25.5)	5.61 (0.74-42.62); *P* = 0.096	4.78 (0.55-41.33); *P* = 0.155
Very high	41 (17.7)	21.04 (2.77-159.77); *P* = 0.003	22.20 (2.45-201.31); *P* = 0.006
IGCCCG			
Good	72 (28.7)	—	—
Intermediate	56 (22.3)	1.97 (0.98-3.97); *P* = 0.058	1.62 (0.70-3.80); *P* = 0.262
Poor	123 (49.0)	2.34 (1.27-4.32); *P* = 0.006	1.28 (0.57-2.88); *P* = 0.547

HR, hazard ratio; IGCCCG, International Germ Cell Cancer Cooperative Group; IPFSG, International Prognostic Factors Study Group; SD, standard deviation.

### Toxicity

Toxicity data under HD-CTX were available for 136 patients in group A (86%) and 86 patients (68%) in group B. Adverse events (other than hematologic) grade ≥3 were reported in 78% in group A and 97% in group B (*P* < 0.001) and treatment-associated deaths in 2% in group A versus 7% in group B (*P* = 0.08). The most frequently stated nonhematological toxicities were neutropenic fever, mucositis, hearing loss, diarrhea, sepsis, and sensoric peripheral neuropathy.

## Discussion

For refractory or recurrent metastatic GCC, the current treatment guidelines recommend salvage CTX, with either CDRs or HD-CTX. When further relapse/progression occurs after HD-CTX, treatment regimens such as GO(P) are frequently used with a palliative intent.^16^, ^17^, ^18^ However, when relapse/progression occurs after CDRs, further salvage treatment using HD-CTX remains a treatment option with curative potential. Nevertheless, only minimal data are available regarding the outcome of patients receiving HD-CTX as a subsequent salvage regimen after prior CDR.

The only comprehensive analysis available to systematically address this question, conducted by Lorch et al.,^14^ revealed disappointing results, with a 5-year OS rate of only 17%, despite an initial response rate of 55%. However, data were collected from several HD-CTX approaches, many of which cannot be now considered standard of care. By contrast, Einhorn et al.^8^ reported findings from a single-center experience where 22 out of 49 patients (44.9%) remained disease free after HD-CE when administered as the third or later line of treatment. Both analyses had a median follow-up time of 48 months.

We hypothesized that salvage treatment with HD-CTX, following at least one conventional salvage treatment line, still possesses curative potential, resulting in long-term remissions for a substantial proportion of patients, albeit likely inferior when compared with the efficacy of HD-CTX as the initial salvage treatment. After establishing an international registry, we included 124 patients who received HD-CTX after at least one salvage treatment line and 159 patients treated with HD-CTX in the first salvage setting. With a 2- and 5-year OS rate of 53% and 37%, respectively, for patients from the initiation of HD-CTX after at least one prior treatment line of CDR, our findings confirm that HD-CTX remains a viable curative treatment option in this context. Here it seems reasonable that patients with prior salvage treatment regimens experience an impaired outcome concerning OS and ORR, as these patients either acquired resistance mechanisms as a result of their exposure to prior CTX regimens or as a result of a selection bias in group B, as this cohort comprises patients in need of further salvage treatment regimens after CDR. Concerning toxicity data, our results revealed that HD-CTX was associated with more toxicity when conducted after CDR. This may be because the cumulative effect of therapies eventually accumulates in the side-effects.

One limitation of this study is its inability to directly compare HD-CTX with CDR as the initial salvage treatment because patients who had a long-term response to a salvage CDR are not included in group B. Group B also encompasses the delivery of HD-CTX across a spectrum of later lines of therapy, as there is no standardized HD-CTX treatment regimen. Furthermore, the missing follow-up is relevantly larger in group B which might influence the OS data.

As a retrospective dataset, we also acknowledge that our study is constrained by data gaps and potential selection bias. Overall, it would be desirable to have prospectively validated markers to predict when CDR alone would be sufficient for patients and when HD-CTX would be necessary.
