# Supplementary material for: Efficacy and safety of high-dose chemotherapy as the first or subsequent salvage treatment line in patients with relapsed or refractory germ cell cancer: an international multicentric analysis
Source: ESMO Open. 2024 May 13;9(5):103449. doi: 10.1016/j.esmoop.2024.103449 (PMC11108831; doi:10.1016/j.esmoop.2024.103449)
Supplement: Supplementary Material [file mmc1.docx]

**Appendix: *Treatment regimens administered.***

| **Treatment** | **Group A**, N = 159^1^ | **Group B**, N = 124^1^ | N=283 (100%) |
| --- | --- | --- | --- |
| **First line** |  |  |  |
| BEP | 135 | 105 | 240 (85%) |
| VIP | 15 | 8 | 23(8%) |
| EP | 2 | 4 | 6(2%) |
| Others  **Second line**  HD-CE  HD-VIP  TIP  VIP  VeIP  Others  **Third line**  TIP  GOP  GO  HD-CE  HD-ICE  HD-CET  Others  **Fourth line**  TIP  GO(P)  HD-CE  HD-TIC  HD-CEC  Others    **Fifth line**  TIP  GO(P)  HD-CE  HD-ICE  Others | 7  158  1  -  -  -  -  4  16  11  -  -  -  -  9  -  -  -  -  -  -  2  -  -  - | 7  -  45  50  17  13  6  82  6  11  19  2  19  2  2  2  4  2  2  1  1 | 15(5%)  158 (56%)  1 (0%)  45 (16%)  50 (18%)  17 (6%)  6 (2%)  10 (4%)  16 (6%)  11 (4%)  82 (29%)  6 (2%)  11 (4%)  19 (7%)  9 (3%)  2 (0,7%)  19 (7%)  2 (0,3%)  2 (0,7%)  2 (0,7%)  4 (1%)  4 (1%)  2 (0,7%)  2 (0,3%)  1 (0,3%) |

**Abbreviations:** BEP: Bleomycine, etoposide, cisplatin; VIP: Etoposide, ifosfamide, cisplatin; HD-CE: High dose carboplatin etoposide; TIP: Paclitaxel, ifosfamide, cisplatin; VeIP: Vinblastin, ifosfamide, cisplatin; HD-CEC: High dose carboplatin, etoposide and cyclophsphamide; HD-ICE: High dose etoposide, ifosfamide, and carboplatin; GOP: Gemcitabine, oxaliplatin and pacitaxel; HD-TIC: High dose paclitaxel, ifosfamide and carboplatin, HD-CET: High dose carboplatin, etoposide and cyclophosphamide, HD-CET: High dose carboplatin, etoposide and thiotepa
